# *Mycobacterium helveticum* sp. nov., a novel slowly growing mycobacterial species associated with granulomatous lesions in adult swine

**DOI:** 10.1099/ijsem.0.004615

**Published:** 2020-12-23

**Authors:** Giovanni Ghielmetti, Giuliana Rosato, Alberto Trovato, Ute Friedel, Constanze Kirchgaessner, Carmen Perroulaz, Wolfgang Pendl, Bettina Schulthess, Guido V. Bloemberg, Peter M. Keller, Roger Stephan, Enrico Tortoli

**Affiliations:** ^1^​ Institute for Food Safety and Hygiene, Section of Veterinary Bacteriology, Vetsuisse Faculty, University of Zurich, Zurich, Switzerland; ^2^​ Institute of Veterinary Pathology, Vetsuisse Faculty, University of Zurich, Zurich, Switzerland; ^3^​ Emerging Bacterial Pathogens Unit, IRCCS San Raffaele Scientific Institute, Milano, Italy; ^4^​ Institute for Infectious Diseases, University of Bern, Bern, Switzerland; ^5^​ Department for Farm Animals, Division of Swine Medicine, Vetsuisse Faculty, University of Zurich, Zurich, Switzerland; ^6^​ Institute of Medical Microbiology, University of Zurich, Zurich, Switzerland; ^7^​ Institute for Food Safety and Hygiene, Swiss National Centre for Enteropathogenic Bacteria and Listeria, Vetsuisse Faculty, University of Zurich, Zurich, Switzerland

**Keywords:** *Mycobacterium helveticum*, NTM, nontuberculous mycobacteria, Switzerland, swine

## Abstract

The occurrence of nontuberculous mycobacteria in different hosts and their implication as obligate or opportunistic pathogens remain mainly unclear. Mycobacteriosis in pigs is usually associated with members of the *Mycobacterium avium* complex and, in particular, with ‘* Mycobacterium avium* subsp. *hominissuis *’. Here we describe a novel slow-growing mycobacterial species isolated from lymph nodes obtained from two sows housed in different Swiss farms. The animals presented chronic inappetence and mild diarrhoea. Gross pathology revealed focal caseous lymphadenopathy of the mesenteric lymph nodes. Complete genome sequencing of the two isolates from the two sows was performed. The genomes comprised 5.76 Mb and an average nucleotide identity score of 99.97 %. Whole genome sequence, mycolic acid and matrix-assisted laser desorption ionization-time of flight mass spectrometry analyses revealed that the two isolates were not related to any previously described *Mycobacterium* species. The closest related species was *Mycobacterium parmense *, a slow-growing scotochromogenic mycobacterium first isolated from a cervical lymph node of a 3-year-old child. The name proposed for the new species is *Mycobacterium helveticum* sp. nov. and 16-83^T^ (=DSM 109965^T^= LMG 2019-02457^T^) is the type strain.

At the time of this writing, the genus *Mycobacterium* contains 207 species and 22 validly published subspecies summarized in the List of Prokaryotic Names with Standing in Nomenclature (www.bacterio.net) [1]. Mycobacteria are aerobic bacteria, non-spore-forming and non-motile. Because of the high concentration of mycolic acid in their cell walls, mycobacteria are difficult to stain with common techniques, such as the Gram stain, therefore alternative staining methods such as Ziehl–Neelsen are commonly used [2]. Among these, several major human and animal pathogens including members of the *Mycobacterium tuberculosis* complex [3] and *Mycobacterium avium* subspecies *paratuberculosis *, the etiological agent of Johne’s disease [4], a contagious disease listed by the World Organisation for Animal Health can be enumerated. Apart from pathogens such as those mentioned above, the presence of nontuberculous mycobacteria (NTM) in different hosts (animals and human) and their implication as obligate or opportunistic pathogens remain mainly ill-defined. Although numerous NTM are harmless to most individuals, an increasing trend in NTM infections, both in veterinary and human medicine, has been observed over the last decades [5–7]. The large majority of NTM species are considered to be ubiquitous and infections occur by ingestion of contaminated water, food or aerosols [8].

Two NTM strains (16-83^T^ and 17–773) were cultured from adult sows both 2 years old presenting granulomatous lesions in the mesenteric lymph nodes. The animals were housed in two different Swiss farms located approximately 10 km apart and, in consequence of chronic inappetence and episodes of diarrhoea, were euthanized in January 2016 and May 2017, respectively. Animal movements between the farms were not reported by the two farmers.

A full necropsy of the first sow, from which the type strain was isolated, was performed. The animal presented tuberculosis-like lesions in the abdomen, characterized by marked caseous lymphadenopathy of the mesenteric lymph nodes. Histologically, the lesions of the lymph nodes revealed a pattern of central necrosis, partially surrounded by dystrophic calcifications. Numerous epithelioid macrophages and multinucleated Langhans giant cells were observed. No acid-fast bacilli could be observed by Ziehl–Neelsen (ZN) staining of the affected lymph nodes.

The second sow suffered from mild diarrhoea and chronic inappetence over a 3 month period and deworming did not lead to improvement of the clinical condition. Since a remarkable number of slaughtered piglets originating from the same farm presented lesions compatible with mycobacteriosis, the sow was euthanized for further diagnostic investigations. The entire intestine, mesenteric lymph nodes, spleen and tonsils were investigated macroscopically and histologically. The mesenteric lymph nodes were enlarged and displayed round lesions with central coagulative necrosis. Numerous epithelioid macrophages and multinucleated Langhans giant cells were observed in the centre of the lesions. Furthermore, in the periphery of the granulomas, several lymphocytes and plasma cells were conglomerated. Histologically, scarce acid-fast bacilli were visible after ZN staining.

The samples were subsequently processed in accordance with standard procedures [9]. Direct microscopy of the homogenized lymph nodes showed low-density of acid-fast rod-shaped bacilli (five to ten bacilli in 300 observed fields) after ZN stain. Growth rate was slow with approximatively 7 weeks in liquid media (BBL MGIT; Becton Dickinson (BD), Sparks, MD) and no growth could be achieved on solid media, e.g. Stonebrink and Löwenstein–Jensen (BD). Subcultures on Middlebrook 7H10 agar plates and Stonebrink slants presented scotochromogenic colonies after three to 4 weeks of incubation at 37 °C. Among the tested culture media, MGIT PZA Medium (BD) with a reduced pH of 5.9 supported the fastest growth (3 weeks to positivity) of the mycobacterium. Growth occurred at temperatures between 25 and 37 °C, while no growth was observed at 40 °C and on MacConkey agar plates without crystal violet at 37 °C.

The whole genome sequences of both clinical strains were obtained as previously described [10]. Briefly, reads were produced by the Illumina platform using Nextera reagents according to the manufacturer’s protocol. Subsequently, quality trimming with TrimGalore and assembling using SPAdes version 3.12 were performed [11]. The assembled genomes were quality controlled with quast version 4.2 [12] and annotated by resorting to the National Center for Biotechnology Information (NCBI) Prokaryotic Genome Annotation Pipeline [13].

The annotated draft genome of type strain 16-83^T^ comprises 5 769 598 bp (NZ_VMQU00000000.1), characterized by 5402 predicted protein-coding sequences and a G+C content of 68.66 mol%. A total of three rRNAs and 56 tRNAs were identified, respectively. Following calculation of average nucleotide identity (ANI) against each of the species of the genus *Mycobacterium* with genome available in public repositories, a genome-based phylogeny was reconstructed from the distance matrix of ANI-divergence according to the UPGMA algorithm (Fig. 1). The ANI score between the two isolates was 99.97 %, indicating a high similarity degree consistent with intra-species variability. The closest related described species was *Mycobacterium parmense* (ANI 84.19 %); a micro-organism first isolated from a cervical lymph node of a 3-year-old child [14]. The *Mycobacterium parmense* DSM 44553^T^ comprises 5 952 912 bp, characterized by 5477 predicted protein-coding sequences and a G+C content of 68.39 %. A total of three rRNAs and 48 tRNAs were described, respectively. The GenBank deposit is SAMN04216945.

**Fig. 1.**
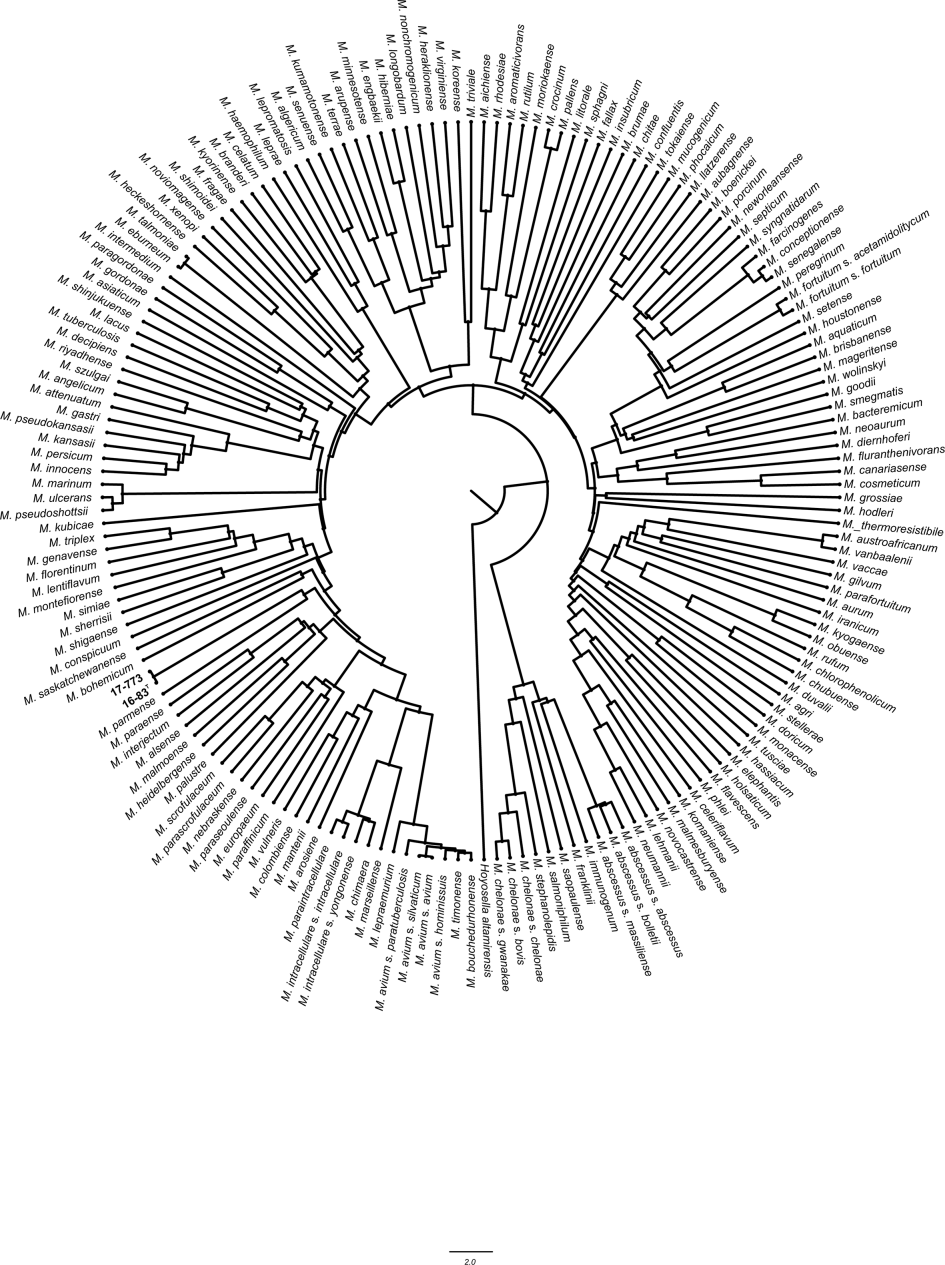
Phylogenetic positioning of strains 16-83^T^ and 17-773 within the genus *Mycobacterium* based on whole genome sequence analysis. Tree reconstructed using the UPGMA algorithm, from the distance matrix of 15 051 ANI divergence scores. Bar, 2 units difference in ANI divergence value. *Hoyosella altamirensis* was used as outgroup.

The three housekeeping genes 16S rRNA, *hsp65* and *rpoB* are among the most common genomic loci used in molecular identification of rapid- and slow-growing mycobacteria [15]. Because the inter- and subspecies similarities at single gene level vary between members of the genus *Mycobacterium *, investigators need to be cautious when isolates are identified based exclusively on single genes. The two strains shared identical 16S rRNA (GenBank MT133249) and *rpoB* (720 bp) gene sequences while the *hsp65* sequences (440 bp) differed by 2 bp (level of similarity, 99.54 %). Sequences of closely related *Mycobacterium* species were retrieved from the NCBI GenBank, aligned with ClustalW [16] and trimmed to start and end at the same nucleotide position [10]. The neighbor-joining method using the Tamura–Nei distance model [17, 18] with 1000 bootstrap replicates was used to reconstruct phylogenetic trees based on the 16S rRNA, *hsp65* and *rpoB* gene sequences (Figs S1–S3, available in the online verion of this article). For both clinical isolates, the three housekeeping gene sequences were divergent from all other available species. A 16S rRNA gene similarity matrix including strain 16-83^T^ and other closely related species was constructed (Fig. S4). Comparisons with 42 different validly published mycobacterial 16S rRNA gene sequences available revealed the highest degree of relatedness with *M. florentinum* (level of similarity, 98.69 %), followed by *M. triplex* and *M. heidelbergense* (both 98.56 %), *M. stomatepiae* (98.49 %) and *M. parmense* (98.42 %). The most closely related species based on the *hsp65* gene sequence was *M. bohemicum* and for *rpoB M. colombiense*, levels of similarity, 97.64 and 92.49%, respectively. A concatenated phylogenetic tree using sequences of the three housekeeping genes 16S rRNA, *rpoB* and *hsp65* was reconstructed and included into the supplementary materials (Fig. S5). The closest related described species included into the phylogenetic analysis was *M. genavense *. The above-mentioned phylogenetic trees based on 16S rRNA gene sequences indicate that the two isolates are distinguishable from their closest species belonging to the *Mycobacterium simiae* complex. The 16S rRNA gene sequence of the 16-83^T^ and 17-773 showed the specific genetic signature present in all the slowly growing species of the complex, including *M. parmense *, *M. paraense *, *M. interjectum* and *M. florentinum* [19]. More specifically, members of the *M. simiae* complex share a 12 bp deletion (short helix) in the hypervariable region 18 (V3) starting at *Escherichia coli* position 459 [20]. Further intrinsic sequence characteristics such as G+C content (68.66 mol%) and total genome length of approximatively 5.8 Mb support the phylogenetic placement of *Mycobacterium helveticum* sp. nov. within the *M. simiae* complex [19].

Although phylogenetic analyses based on 16S rRNA gene and whole genome sequence data partially confirmed the degree of relatedness between mycobacterial species [19], recent studies suggest that ANI may be superior to a single gene for measuring genetic relatedness, and should not be prone to varied evolutionary rates or horizontal gene transfer events [21, 22]. Closely related species based on the 16S rRNA gene sequences analysis such as *M. florentinum, M. triplex* and *M. heidelbergense *, displayed a lower ANI value compared to *M. parmense *, 80.76, 83.03 and 81.28 %, respectively. Discrepancies between the topology of phylogenetic trees based on single housekeeping genes (Figs S1–S3) compared to concatenated sequences (Fig. S5) or whole genome sequences (Fig. 1) appear evident for the tested strains as well as for species of the *M. simiae* complex such as *M. europaeum* [20], *M. saskatchewanense* [23] and the newly described *M. rhizamassiliense* and *M. numidiamassiliense* [24]. In particular, these findings further support previous observations on members of this complex, for which single-gene based classification potentially leads to phylogenetical misplacement [19].

Comparison search based on the assembled genome of type strain 16-83^T^ using PathogenFinder (https://cge.cbs.dtu.dk/services/PathogenFinder/) predicted the new species to be a potential human pathogen with a probability of 0.82, while *Mycobacterium tuberculosis* H37Rv Siena possess a predicted probability of 0.92 [25].

For major biochemical reactions recommended for the speciation of mycobacteria, both strains were grown on Middlebrook 7H10 agar plates for 4 weeks at 37 °C. Previously described methods were used to determine catalase and urease activity, nitrate and tellurite reduction, and Tween 80 hydrolysis [26]. Semi-quantitative and thermostable catalase (pH 7, 68 °C) tests were positive and both strains exhibited tellurite reduction, whereas nitrate reduction, Tween 80 hydrolysis (10 days) and urease activity tested negative (Table 1). These biochemical features distinguished the two strains from closely related species which have urease (* M. parmense* [14] and *M. interjectum* [27]) and Tween 80 hydrolysis activity (* M. parmense* [14]) or do not reduce tellurite (* M. parmense* [14] and *M. paraense* [28])

**Table 1.** Phenotipic characteristics of strains 16-83^T^ and 17-773 in comparison with the closely related species Strains: 1, 16-83^T^; 2, 17-773; 3, *Mycobacterium parmense* [14]; 4, *Mycobacterium paraense* [28]; 5, *Mycobacterium interjectum* [27]; S, scotochromogenic; +, positive; −, negative.

Characteristics	1	2	3	4	5
Pigmentation	S	S	S	S	S
Growth at 25 °C	+	+	+	+	−
Growth at 42 °C	−	−	−	−	−
Semi-quantitative catalase	+	+	−	+	+
Thermostable catalase	+	+	+	+	+
Nitrate reduction	−	−	−	−	−
Urease	−	−	+	−	+
Tween 80 hydrolysis	−	−	+	−	−
Tellurite reduction	+	+	−	−	−

High-performance liquid chromatography (HPLC) profiles of cell-wall mycolic acids of the type strain and the closely related *Mycobacterium parmense* DSM 44553^T^ (German Collection of Microorganisms and Cell Cultures) were obtained as recently described [29–31] following the recommendations of the Sherlock Mycobacteria Identification System [32]. Briefly, cells were grown in MGIT liquid medium (BD), saponified, extracted and derivatized. Mycolic acids were separated with a gradient of methanol and 2-propanol on an Agilent ChemStation 1100/1200 HPLC system. Peak integration and identification were performed using the MIDI Sherlock Software version 6.2B. Strain 16-83^T^ produced a unique mycolic acid profile characterized by two clusters of three major peaks eluting between 3–4 min and 7–8 min. This profile is clearly different from the one obtained from *Mycobacterium parmense* DSM 44553^T^ (Fig. 2). According to the Sherlock software, the closest available profile belonged to a rare variant of *Mycobacterium gordonae* with a similarity index of 0.744.

**Fig. 2.**
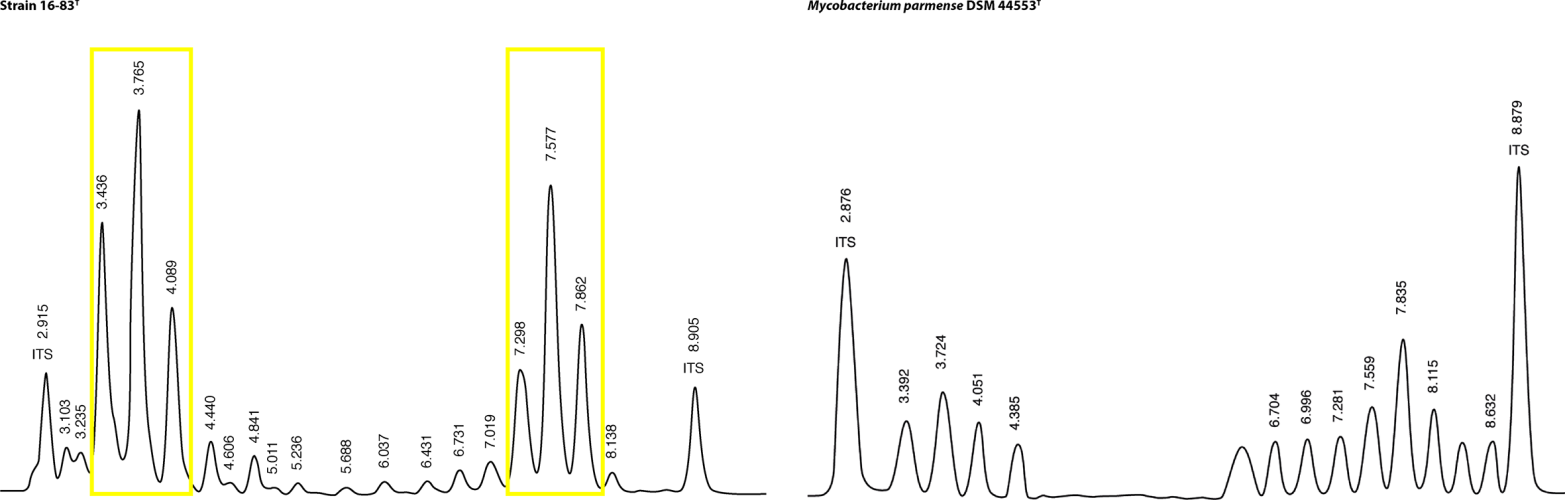
Representative mycolic acid pattern of strain 16–83^T^ in comparison to *Mycobacterium parmense* DSM 44553^T^. Strain 16-83^T^ produced a specific mycolic acid profile characterized by two clusters of three major peaks eluting between 3–4 min and 7–8 min (yellow boxes). Numbers indicate retention times (minutes). ITS, internal standard.

A total of five matrix-assisted laser desorption ionization-time of flight (MALDI-TOF) mass spectrometry (MS) profiles for each of the two isolates were acquired on a Bruker microflex system and interpreted using the Bruker mycobacterial database (version 4.0). Inactivation and preparation of the isolates for MALDI‐TOF MS analysis were performed using the Mycobacteria Extraction Method (MycoEX) in accordance with the manufacturer and as previously described [33]. Solid Middlebrook 7H10 agar plates incubated at 37 °C were chosen as culture medium and biomass was harvested approximatively 4 weeks after inoculation. The profiles were obtained from five different colonies and two technical replicates for each of the two isolates. Routine identification was not possible because a matching main spectral profile (MSP) was not present in the database. The obtained profiles from the two isolates formed one single indistinguishable clade on a dendrogram created using the Biotyper compass version 3.0 software. Ten mass spectra of type strain 16-83^T^ were exported and processed with the MALDIQuantForeign and MALDIQuant packages in R [31]. The obtained averaged spectrum is shown in Fig. 3 and could be implemented as MSP in the mycobacterial database for the identification of further isolates.

**Fig. 3.**
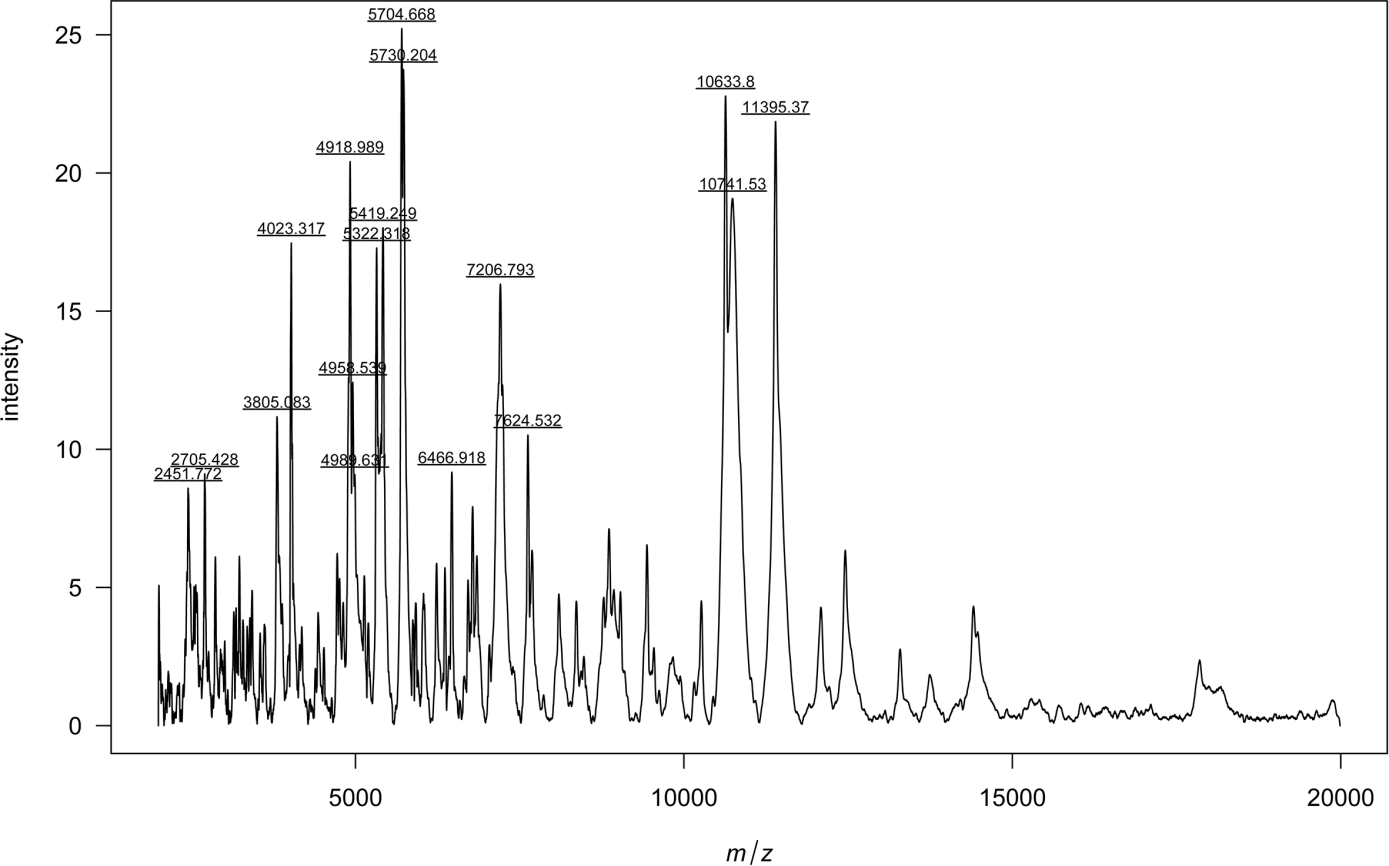
MALDI-TOF MS spectra of strain 16–83^T^. Main spectral profile obtained from ten averaged mass spectra of strain 16–83^T^ imported into R and processed with the MALDIQuantForeign and MALDIQuant packages. m/z represents mass divided by charge number.

Phenotypic susceptibility testing of the two strains was first attempted with a standard commercial broth microdilution method for slowly growing NTM according to the manufacturer’s instructions (SLOMYCO Sensititre, Thermo Fischer Scientific). The recommended Sensititre cation adjusted Mueller–Hinton broth w/TES did not support the growth of both isolates even after a prolonged incubation time of 60 days at 37 °C and was therefore not suitable for this purpose. Antimicrobial susceptibility testing was thereafter performed with a standardized microdilution method for slowly growing mycobacteria as described by the Clinical and Laboratory Standard Institute (CLSI) [34]. The two isolates were grown in MGIT PZA medium (BD) supplemented with mycobactin J and minimal inhibition concentrations were read after 14 days of incubation at 37 °C (Table 2). CLSI breakpoints were used for the interpretation of the MIC data [35]. The isolates were interpreted as susceptible to amikacin, clarithromycin, linezolid, rifabutin, rifampicin and clofazimine. The MIC of moxifloxacin was borderline. Based on the MIC values (>32 mg l^−1^), resistance to isoniazid was considered for both strains; while growth in presence of ethambutol was not interpretable. Minimal inhibition concentrations for the type strain of *M. parmense* DSM 44553^T^ were determined under identical conditions. Except for clarithromycin, for which a higher MIC value was observed (4 mg l^−1^), overall similar MIC values for the antimicrobials included were shown in comparison to strains 16-83^T^ and 17–773.

**Table 2.** MICs of strains 16-83^T^, 17–773 and *Mycobacterium parmense* DSM 44553^T^ to antimycobacterial drugs S, susceptible; I, intermediate; R, resistant according to CLSI breakpoints for nontuberculous mycobacteria.

Antimicrobial	Conc. range tested (mg l^−1^)	MIC (mg l^−1^) observed for strains 16-83^T^ and 17–773	Interpretation	MIC (mg l^−1^) observed for *M. parmense* DSM 44553^T^
Amikacin	0.03–16	2–16	S	16
Clarithromycin	0.25–128	0.5	S	4
Clofazimine	0.03–16	0.25–1	S	0.125
Ethambutol	0.06–32	/	Not interpretable	/
Isoniazid	0.06–32	>32	R	32
Linezolid	0.06–32	1–8	S	8
Moxifloxacin	0.03–16	0.125–2	S-I	0.5
Rifabutin	0.004–2	0.004–0.008	S	0.008
Rifampicin	0.06–32	0.125	S	0.125

Nassociated with a clinical disease may be encountered repeatedly, especially under defined settings like farmed domestic pigs. The fastidious nature of the hereby described isolates and their long generation time on commercial culture media may represent the reason why this mycobacterium has not been isolated before. Histopathological lesions compatible with mycobacteriosis of the digestive tract in sows are not rare [36–39]. Although numerous mycobacteria, e.g. *Mycobacterium fortuitum, Mycobacterium gordonae*, *Mycobacterium terrae *, *Mycobacterium chelonae *, *Mycobacterium smegmatis *, *Mycobacterium phlei* and *Mycobacterium scrofulaceum *, can be involved in the formation of such lesions in domestic pigs, members of the *M. avium* complex are without doubt the most common [40]. ‘* M. avium* subsp. *hominissuis *’ in particular, is held responsible for the large majority of the lesions observed at meat inspection in the abattoirs, and often, no further bacteriological investigations, including species identification, are undertaken. Nevertheless, severe economic losses for farmers, primarily resulting from condemnation of pig meat and visceral organs, are reported [38]. Furthermore, financial penalties can occur from movement restriction of live animals. In countries implementing eradication programmes for bovine tuberculosis and extending their regulations to non-bovine species, farms housing pigs infected with unknown mycobacteria may undergo strict restrictions. It appears therefore crucial that mycobacteria associated with clinical lesions should be further characterized and identified at species level for risk assessment.

## Description of *Mycobacterium helveticum* sp. nov.

*Mycobacterium helveticum* (hel.ve’ti.cum. L. neut. adj. *helveticum* pertaining to Helvetia, the Latin name of Switzerland, from where the two first known strains originated).

Non-motile, non-spore-forming rod-shaped and acid-fast. Growth on solid media requires >3 weeks at temperatures ranging from 25–37 °C. Colonies on Middlebrook 7H10 agar plates and Stonebrink slants are smooth, raised with round or lobate regular margins and scotochromogenic. The species produces >45 mm foam in the semi-quantitative catalase test, is positive for heat-stable catalase and also exhibits tellurite reduction. Reactions for nitrate, Tween 80 hydrolysis and urease activity are negative. No growth was achieved either at 40 °C nor on MacConkey agar without crystal violet. The strains were susceptible to the antimicrobials tested (amikacin, clarithromycin, linezolid, rifabutin, rifampicin and clofazimine) with the exception of isoniazid, for which resistance was observed for both isolates. The MIC of moxifloxacin was borderline.

The annotated draft genome of type strain 16-83^T^ comprises 5 769 598 bp and the 16S rRNA gene sequence are deposited in a public database (GenBank, NCBI) under accession numbers NZ_VMQU00000000.1 and MT133249, respectively. The type strain is 16-83^T^ (=DSM 109965^T^=LMG 2019-02457^T^).

## Supplementary Data

Supplementary material 1
