# Supplementary material for: Mycobacterium helveticum sp. nov., a novel slowly growing mycobacterial species associated with granulomatous lesions in adult swine
Source: Int J Syst Evol Microbiol. 2020 Dec 23;71(1):ijsem004615. doi: 10.1099/ijsem.0.004615 (PMC7968739; doi:10.1099/ijsem.0.004615)
Supplement: Supplementary material 1 [file ijsem-71-615-s001.pdf]

Mycobacterium helveticum sp. nov., a novel slowly growing mycobacterial species associated with granulomatous lesions in adult swine

---

Giovanni Ghielmetti[1,*], Giuliana Rosato[2], Alberto Trovato[3], Ute Friedel[1], Constanze Kirchgaessner[1], Carmen Perroulaz[4], Wolfgang Pendl[5], Bettina Schulthess[6], Guido Bloemberg[7], Peter M. Keller[4], Roger Stephan[1], Enrico Tortoli[3]

[1]Institute for Food Safety and Hygiene, Section of Veterinary Bacteriology, Vetsuisse Faculty University of Zurich, Zurich, Switzerland

[2]Institute of Veterinary Pathology, Vetsuisse Faculty University of Zurich, Zurich, Switzerland

[3]Emerging Bacterial Pathogens Unit, IRCCS San Raffaele Scientific Institute, Milano, Italy

[4]Institute for Infectious Diseases, University of Bern, Bern, Switzerland

[5]Department for Farm Animals, Division of Swine Medicine, Vetsuisse Faculty, University of Zurich, Zurich, Switzerland

[6] Institute of Medical Microbiology, University of Zurich, Zurich, Switzerland

[7]Institute for Food Safety and Hygiene, Swiss National Centre for Enteropathogenic Bacteria and Listeria, Vetsuisse Faculty University of Zurich, Zurich, Switzerland

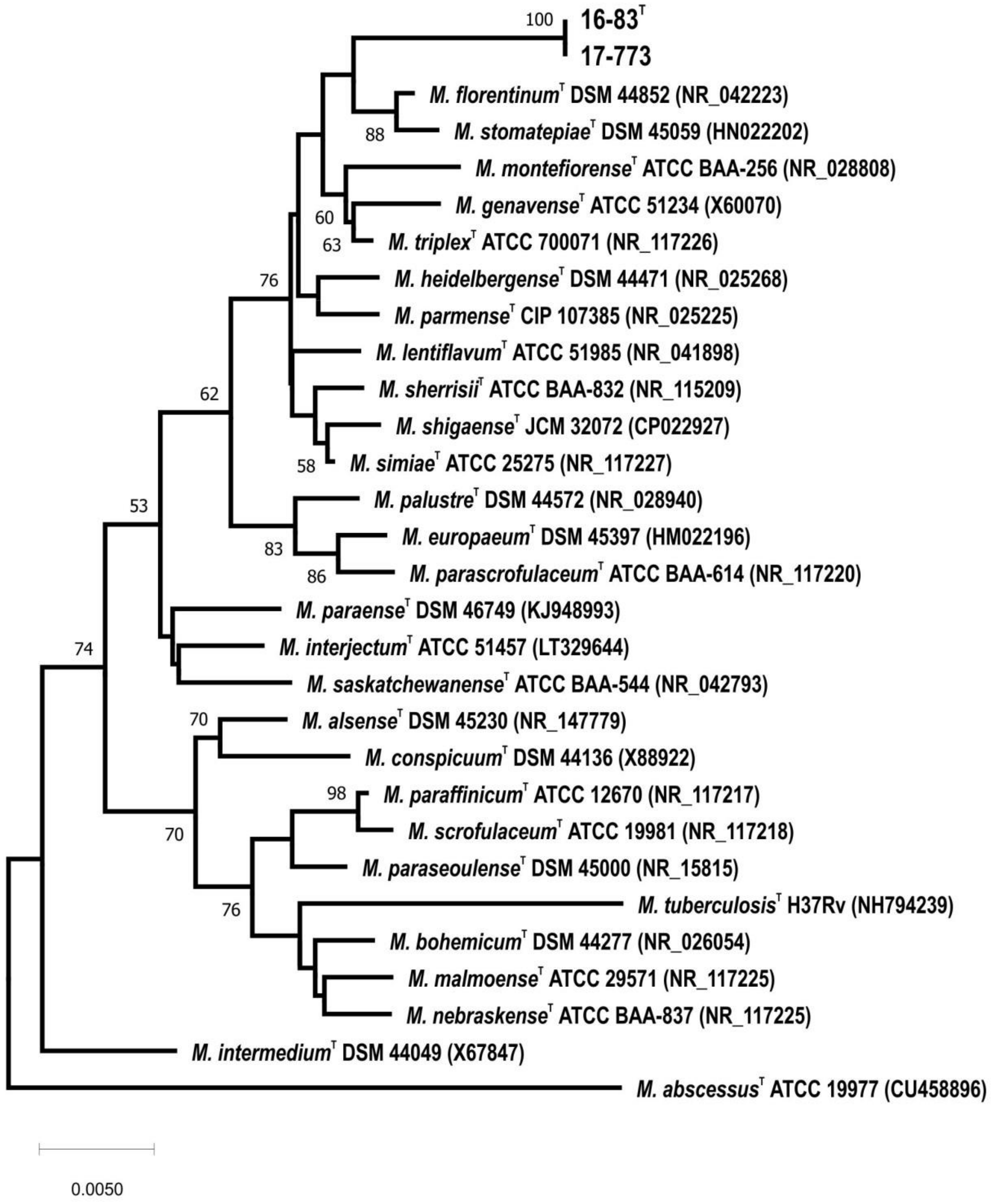

Supplementary Figure S1. Phylogenetic tree based on complete 16S rRNA gene sequences, constructed using the neighbor-joining method bootstrapped 1000 times. Bootstrap values >50 are given at nodes. Bar, 0.005 substitutions per nucleotide position. *M. abscessus*, outgroup.

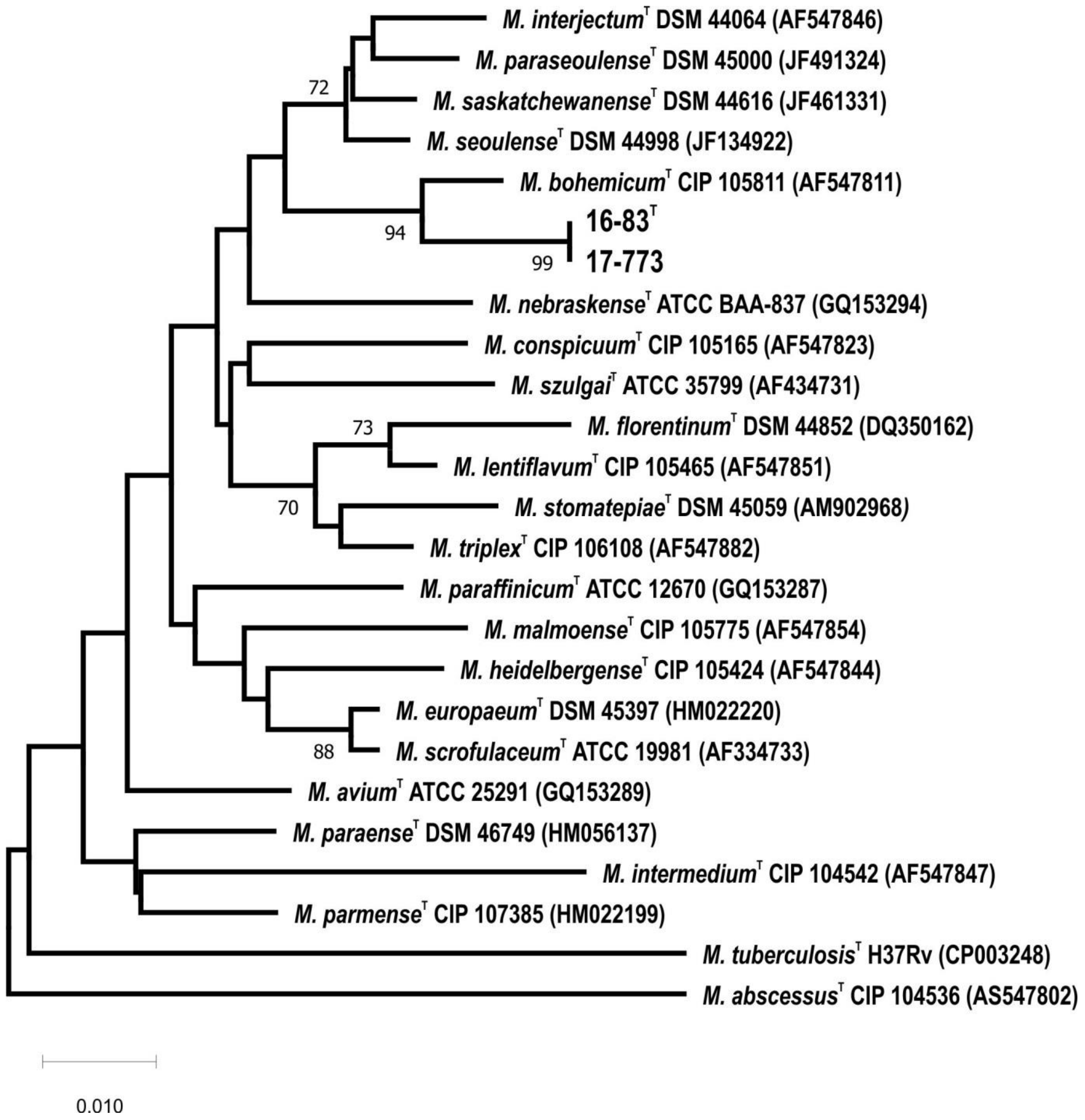

Supplementary Figure S2. Phylogenetic tree based on partial *hsp65* gene sequences, constructed using the neighbor-joining method bootstrapped 1000 times. Bootstrap values >50 are given at nodes. Bar, 0.01 substitutions per nucleotide position. *M. abscessus*, outgroup.

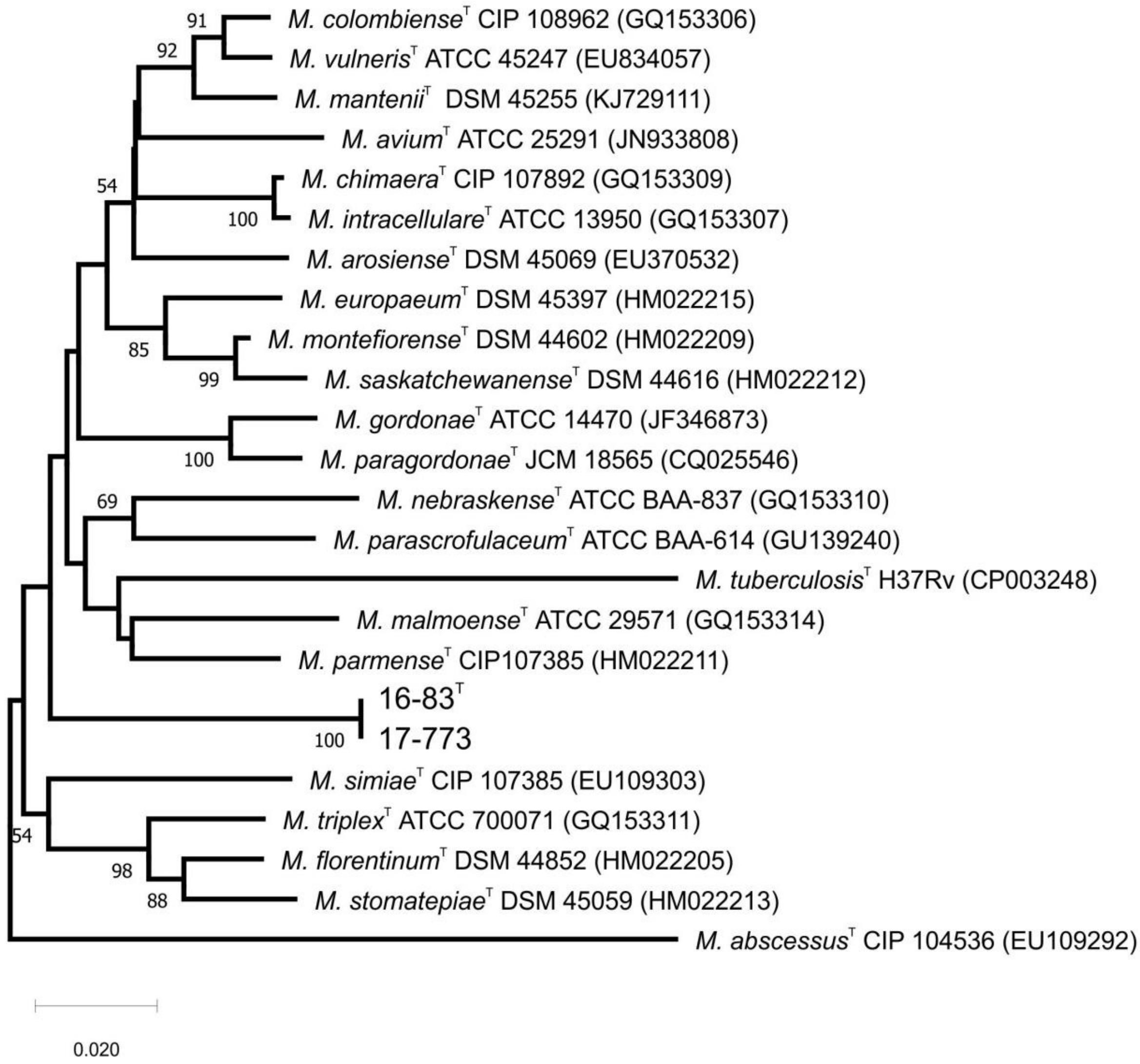

Supplementary Figure S3. Phylogenetic tree based on partial *rpoB* gene sequences, constructed using the neighbor-joining method bootstrapped 1000 times. Bootstrap values >50 are given at nodes. Bar, 0.02 substitutions per nucleotide position. *M. abscessus*, outgroup.

	16-83	M.abscessu	M.alsense	M.arosiens	M.avium	M.bohemic	M.celatum	M.chimaera	M.colombie	M.conspicu	M.europaeu	M.florentinu	M.genaven	M.haemopl	M.heidelbe	M.interjectu	M.intermed	M.intracellu	M.kansasii	M.lentiflavu	M.malmoer	M.mantenii	M.marinum	M.montefio	M.nebrask	M.palustre	M.paraense	M.paraffinic	M.parascro	M.paraseou	M.parmens	M.saskatch	M.scrofulac	M.sherrisii	M.shigaens	M.shimoide	M.simiae	M.stomatep	M.triplex	M.tuberculo	M.ulcerans	M.vulneris	M.xenopi
16-83		95.39	96.72	95.98	96.25	96.39	95.57	96.05	96.18	96.04	98.21	98.69	98.07	95.91	98.56	97.80	97.18	96.18	95.77	98.21	96.12	96.45	96.39	98.21	96.05	98.07	97.80	96.45	98.08	96.25	98.42	98.00	96.31	98.21	98.28	95.10	98.21	98.49	98.56	95.98	96.39	96.18	93.77
M.abscessus			94.54	95.22	95.02	94.68	93.95	94.81	95.15	94.74	96.01	95.52	95.59	94.68	95.32	95.66	96.28	94.95	94.47	95.39	94.47	95.21	94.81	95.80	94.60	95.80	95.80	95.21	95.66	94.87	95.39	95.60	95.07	95.52	95.59	94.41	95.59	95.39	95.66	94.40	94.67	95.01	92.81
M.alsense				98.50	98.57	98.77	97.21	98.43	98.36	99.11	96.93	97.00	96.79	98.71	97.47	98.23	97.28	98.64	98.36	97.13	98.98	98.16	98.02	96.79	98.71	97.27	97.82	98.23	96.66	98.91	97.00	97.75	98.30	97.47	97.20	97.08	97.47	97.07	97.13	97.89	98.02	98.43	95.05
M.arosiense					99.32	98.91	96.60	99.45	99.73	98.23	97.20	96.52	96.18	98.64	96.39	97.14	97.54	99.66	98.50	96.86	98.57	98.64	98.09	96.45	98.70	97.27	97.28	98.50	96.93	99.04	96.52	97.48	98.57	96.59	96.59	97.21	96.73	96.39	96.52	97.75	97.95	99.52	95.19
M.avium						99.11	96.60	99.05	99.59	98.23	97.00	96.39	96.46	98.91	96.59	97.21	97.27	99.25	98.70	96.73	98.84	98.77	98.43	96.59	98.77	97.07	97.14	98.30	97.00	98.84	96.73	97.41	98.36	96.46	96.32	97.28	96.53	96.12	96.80	98.16	98.43	99.52	94.99
M.bohemicum							96.46	98.84	99.04	98.36	97.34	96.46	96.39	99.25	97.14	97.48	97.48	99.04	99.11	96.66	99.32	98.70	98.64	96.66	99.32	97.20	97.07	98.64	97.61	99.18	96.87	97.96	98.70	96.80	96.39	97.14	96.66	96.46	96.73	98.36	98.64	98.98	94.85
M.celatum								96.53	96.60	96.94	95.71	96.12	95.64	96.40	95.57	96.12	95.58	96.67	96.47	95.71	96.60	96.47	96.80	95.58	96.54	95.78	96.39	96.68	95.44	96.81	95.64	95.92	96.67	95.78	95.92	97.14	95.92	95.91	95.85	96.81	96.80	96.47	95.80
M.chimaera									99.32	98.16	97.27	96.18	95.84	98.77	96.32	97.14	97.47	99.80	98.57	96.52	98.63	98.57	98.09	96.11	98.70	97.27	97.21	98.64	97.00	99.04	96.32	97.48	98.70	96.39	96.39	97.07	96.52	96.04	96.18	97.75	98.02	99.39	94.99
M.colombiense										98.09	97.06	96.52	96.45	98.77	96.52	97.00	97.27	99.52	98.63	97.00	98.70	98.77	98.36	96.72	98.84	97.13	97.21	98.36	97.06	98.91	96.80	97.61	98.43	96.45	96.53	97.07	96.59	96.25	96.79	98.02	98.23	99.80	94.99
M.conspicuum											97.00	96.52	96.38	98.36	96.79	97.54	97.07	98.36	98.29	96.86	98.43	97.95	97.95	96.72	98.50	97.13	97.47	98.16	96.72	98.70	96.79	97.20	98.23	97.00	97.00	97.07	97.27	96.59	96.66	97.75	97.95	98.16	95.19
M.europaeum												98.49	98.21	97.07	98.62	98.21	98.49	97.40	97.27	98.69	97.06	97.67	96.52	98.42	97.40	99.31	98.14	98.22	99.59	97.88	98.76	98.28	98.08	98.83	98.83	96.19	99.11	98.49	98.49	96.32	96.45	97.06	94.18
M.florentinum													99.11	96.18	98.76	98.28	97.46	96.32	95.91	98.97	96.25	96.52	96.32	99.11	96.18	98.42	98.42	96.86	98.21	96.52	98.90	97.94	96.72	99.38	99.31	95.78	99.24	99.73	99.45	96.12	96.18	96.39	94.25
M.genavense														96.32	98.83	98.07	97.32	95.98	96.04	99.11	96.32	96.52	95.98	99.04	96.18	98.22	98.21	96.58	98.21	96.25	99.04	97.87	96.45	99.17	99.11	95.37	99.11	98.83	99.52	95.78	95.84	96.45	93.56
M.haemophilum															97.14	97.55	97.47	98.84	98.98	96.80	99.39	98.36	98.50	96.66	99.18	97.27	97.14	98.43	97.34	98.84	96.80	97.62	98.50	96.66	96.52	97.21	96.73	96.18	96.59	98.30	98.43	98.98	94.79
M.heidelbergense																98.69	98.01	96.45	96.86	99.24	97.27	96.93	96.32	98.83	97.00	98.83	98.28	96.99	98.90	96.79	99.31	98.76	96.86	99.24	98.97	95.37	99.24	98.76	99.17	96.12	96.18	96.66	93.56
M.interjectum																	98.63	97.27	97.00	98.49	97.41	97.40	96.52	98.07	97.14	98.49	99.18	97.81	98.08	97.48	98.28	99.11	97.81	98.76	98.49	95.99	98.76	98.28	98.42	96.39	96.52	97.14	94.17
M.intermedium																		97.61	97.68	97.87	97.47	97.61	96.93	97.39	97.41	98.49	98.35	98.09	98.21	97.75	97.67	98.42	97.95	97.94	97.87	96.19	98.14	97.46	97.67	96.66	96.80	97.41	94.38
M.intracellulare																			98.70	96.66	98.84	98.64	98.16	96.24	98.91	97.41	97.35	98.70	97.13	99.25	96.46	97.61	98.77	96.52	96.53	97.21	96.66	96.18	96.32	97.95	98.09	99.45	95.13
M.kansasii																				96.52	99.18	98.50	98.57	96.11	99.25	96.86	96.93	98.64	97.54	99.11	96.86	97.48	98.63	96.39	96.39	97.00	96.66	95.91	96.32	98.64	98.50	98.70	94.72
M.lentiflavum																					96.66	97.00	95.91	98.90	96.66	99.04	98.62	97.06	98.69	96.72	99.24	98.69	96.92	99.31	99.31	95.51	99.59	98.97	99.38	95.71	95.77	97.00	93.84
M.malmoense																						98.43	98.50	96.59	99.39	97.13	97.00	98.36	97.34	99.04	96.80	97.48	98.43	96.73	96.45	97.07	96.73	96.25	96.66	98.57	98.43	98.77	94.79
M.mantenii																							97.88	96.85	98.77	97.26	97.47	99.38	97.67	98.98	96.93	97.75	99.45	96.72	96.72	97.07	97.00	96.52	96.79	97.55	97.88	98.70	94.79
M.marinum																								95.97	98.50	96.38	96.52	97.89	96.79	98.29	96.52	96.86	97.95	95.98	96.04	97.07	96.04	96.11	96.32	99.11	99.86	98.57	95.13
M.montefiorense																									96.52	98.35	97.80	96.78	98.42	96.58	98.83	97.87	96.65	99.04	98.69	95.44	98.90	98.83	99.38	95.77	95.84	96.59	93.77
M.nebraskense																										97.00	97.07	98.70	97.68	99.39	96.86	97.61	98.77	96.52	96.52	97.21	96.79	96.18	96.45	98.43	98.43	98.77	94.92
M.palustre																											98.42	97.67	99.04	97.34	98.56	98.42	97.54	98.90	98.83	96.05	99.04	98.49	98.56	96.25	96.38	97.27	94.31
M.paraense																												97.75	97.87	97.41	98.21	99.04	97.61	98.42	98.97	96.40	98.69	98.35	98.28	96.39	96.52	97.21	94.72
M.paraffinicum																													97.95	99.18	97.13	97.88	99.80	97.20	97.20	97.21	97.47	96.86	96.86	97.69	97.82	98.43	94.93
M.parascrofulaceum																														97.61	99.04	98.56	97.95	98.56	98.56	95.91	98.83	98.21	98.49	96.59	96.72	97.06	93.91
M.paraseoulense																															96.79	97.54	99.25	96.86	96.86	97.41	97.13	96.52	96.52	98.16	98.23	98.84	95.13
M.parmense																																98.62	97.00	99.11	99.11	95.45	99.38	98.62	99.31	96.25	96.39	96.93	93.77
M.saskatchewanense																																	97.75	98.28	98.21	95.71	98.42	97.94	98.21	96.60	96.86	97.61	94.11
M.scrofulaceum																																		97.06	97.06	97.21	97.33	96.72	96.72	97.68	97.88	98.50	94.93
M.sherrisii																																			99.45	95.58	99.73	99.38	99.52	95.78	95.84	96.59	93.77
M.shigaense																																				95.92	99.73	99.24	99.17	95.85	95.91	96.66	94.11
M.shimoidei																																					95.78	95.65	95.58	96.80	97.07	96.94	97.02
M.simiae																																						99.24	99.38	95.85	95.91	96.73	93.97
M.stomatepiae																																							99.17	95.98	95.98	96.11	94.17
M.triplex																																								95.98	96.18	96.79	93.77
M.tuberculosis																																									99.05	98.09	95.20
M.ulcerans																																										98.43	95.13
M.vulneris																																											94.86

Supplementary Figure S4. 16S rRNA similarity matrix. The accession numbers for sequences obtained from GenBank are as follow: 16-83, MT133249; M. abscessus, CU45889; M. alsense, NR_147779; M. arosiense, NR_117221; M. avium, , CP046507; M. bohemicum, NR_026054; M. celatum, NR_114894; M. chimaera, CP015278; M. colombiense, CP020821; M. conspicuum, X88922; M. europaeum, NR_125568; M. florentinum, NR_042223; M. genavense, X60070; M. haemophilum, X88923; M. heidelbergense, NR_025268; M. interjectum, LT329644; M. intermedium, X67847; M. intracellulare, CP003322; M. kansasii, NR_1210712; M. lentiflavum, NR_041898; M. malmoense, NR_117225; M. mantenii, NR_116538; M. marinum, NR_025214; M. montefiorense, NR_028808; M. nebraskense, NR_117225; M. palustre, NR_028940; M. paraense, KJ948993; M. paraffinicum, NR_117217; M. parascrofulaceum, NR_117220; M. paraseoulense, NR_115815; M. parmense, NR_025225; M. saskatchewanense, NR_042793; M. scrofulaceum, NR_117218; M. sherrisii, NR_115209; M. shigaense, CP022927; M. shimoidei, NR_041945; M. simiae, NR_117227; M. stomatepiae, HN022202; M. triplex, NR117226; M. tuberculosis, NH794239; M. ulcerans, X88926; M. vulneris, LT718448; M. xenopi, NH169241.

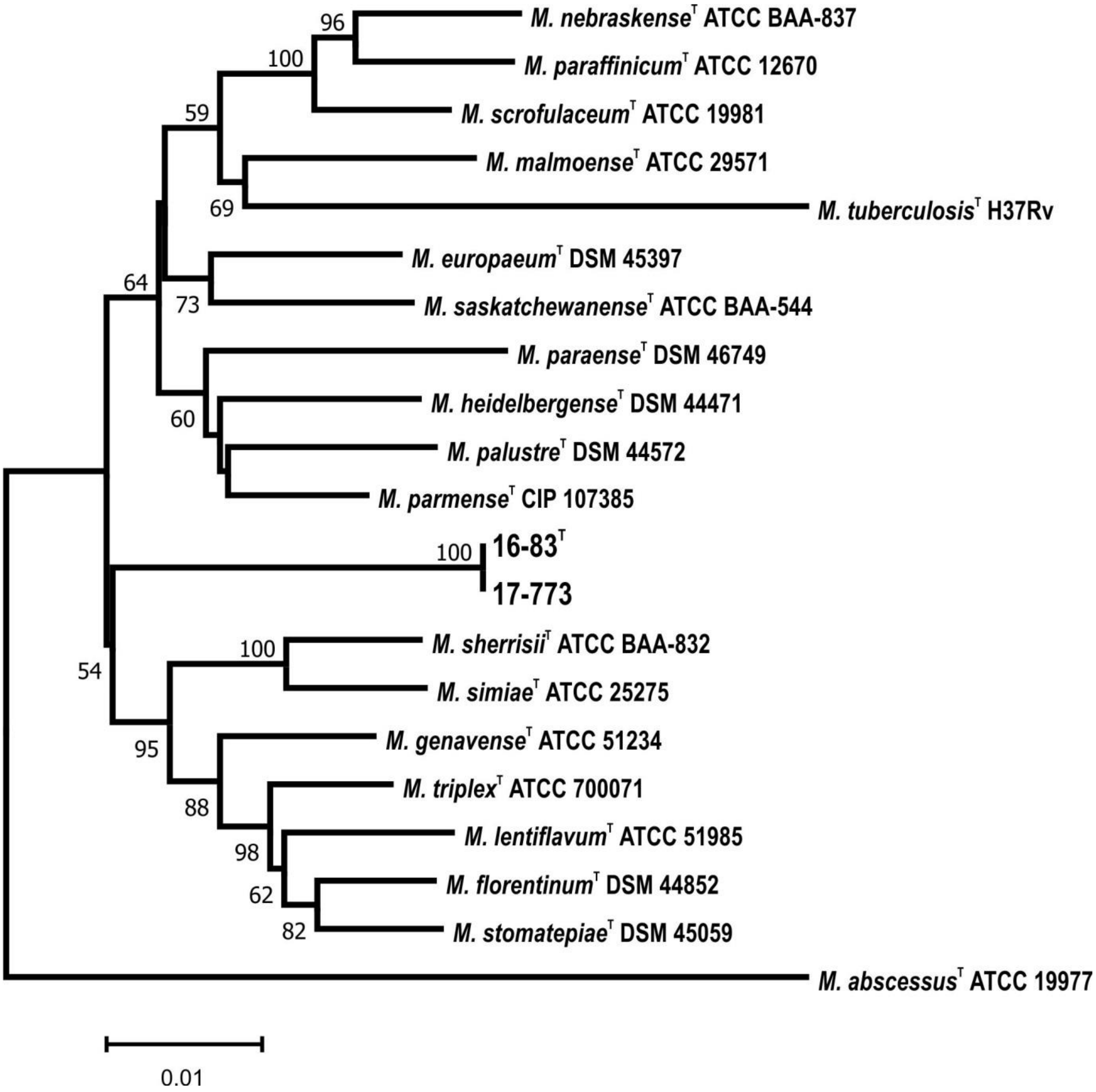

Supplementary Figure S5. Phylogenetic tree based on concatenated sequences of 16S rRNA, *hsp65* and *rpoB* gene sequences of representative species of the genus *Mycobacterium*, reconstructed using the neighbor-joining method bootstrapped 1000 times. Bootstrap values >50 are given at nodes. Bar, 0.01 substitutions per nucleotide position. *M. abscessus*, outgroup.
